# Data Analysis of Infection Control Awareness and Practices Among Healthcare Workers: A Cross-Sectional Study in a Tertiary-Care Hospital in Kashmir, India

**DOI:** 10.7759/cureus.95179

**Published:** 2025-10-22

**Authors:** Fatima Abeer, Aasim Ayaz Wani, Shoaib M Khan, Anjum Farhana

**Affiliations:** 1 Microbiology, Government Medical College, Srinagar, IND; 2 Chemical and Biomolecular Engineering, Cornell University, New York, USA

**Keywords:** cross-sectional study, healthcare-acquired infection, healthcare-associated infections (hcais), healthcare workers, infection prevention and control practices, promoting infectious disease control

## Abstract

Healthcare-associated infections (HAIs) pose a significant threat to patient and healthcare worker (HCW) safety, particularly in low-resource settings. While infection prevention and control (IPC) measures can effectively reduce HAIs, adherence among HCWs often varies. This study assessed IPC awareness and practices among HCWs in a tertiary-care hospital in Kashmir, India, aiming to identify areas for targeted intervention. A cross-sectional study was conducted among 300 HCWs at Government Medical College (GMC), Srinagar, and its associated hospitals using an online questionnaire to assess demographics, comorbidities, IPC knowledge, and self-reported adherence to key practices. While the majority of respondents (93%) reported awareness of IPC measures, adherence to key practices was suboptimal. Regular handwashing was practiced by only 56% of HCWs, while mask use stood at 17% and hand-sanitizer use at 20%. Needlestick injuries were reported by 16% of HCWs, highlighting gaps in standard precautions. Despite substantial IPC awareness, consistent application remains a challenge in this tertiary-care setting. Strengthening training programs, ensuring resource availability, and promoting a culture of safety are crucial to improve compliance, reduce HAIs, and protect both patients and HCWs. These findings underscore the need for targeted interventions to bridge the knowledge-practice gap and enhance IPC effectiveness in similar settings.

## Introduction

Background and significance of healthcare-associated infections

Healthcare-associated infections (HAIs) pose a significant threat to patient safety, causing preventable illness, death, and increased healthcare costs worldwide [1]. Globally, HAIs affect 7-10% of hospitalized patients in high-income countries and up to 15% in low- and middle-income countries (LMICs), where resource limitations exacerbate the risks [2]. This disparity highlights the disproportionate burden faced by resource-constrained settings. HAIs contribute to significant morbidity and mortality, with an estimated 1 in 10 patients affected by an HAI dying from their infection [3]. In India alone, HAIs contribute to over 2 million infections annually, with mortality rates exceeding 10-30% and prolonged hospital stays costing an estimated $1.5 billion each year [4]. The COVID-19 pandemic further exposed systemic weaknesses in infection control, as hospitals struggled with increased nosocomial outbreaks [5]. Despite being largely preventable through evidence-based measures, HAIs remain a critical public health concern, particularly in LMICs like India [6]. Overcrowding, understaffing, and fragmented supply chains create significant challenges to consistent adherence to infection prevention and control (IPC) protocols. Healthcare workers (HCWs) are at the forefront of these efforts, playing a crucial role in preventing the transmission of HAIs. However, they often face numerous challenges, including heavy workloads, limited resources, and systemic barriers, which can hinder their ability to consistently adhere to optimal IPC protocols.

Importance of IPC measures

Effective IPC measures are essential to minimizing HAIs, protecting vulnerable patients, and ensuring the safety of HCWs [1]. It is estimated that 70% of HAIs be prevented with good hand hygiene and other cost-effective practices [7]. Consistent hand hygiene alone can decrease HAI incidence by up to 50%, while appropriate use of personal protective equipment (PPE) and thorough environmental disinfection further reduce transmission risks [5]. Unfortunately, global adherence to these critical practices remains suboptimal. Fewer than 40% of HCWs in LMICs consistently comply with hand hygiene protocols, and PPE shortages continue to plague even tertiary-care facilities [8]. While the COVID-19 pandemic temporarily increased awareness and adoption of practices like mask-wearing and isolation protocols, evidence suggests that these improvements are often short-lived [9]. Studies, such as the one by Anderson-Carpenter and Tacy, demonstrate that post-pandemic hand hygiene compliance in Indian hospitals frequently returns to pre-COVID levels within six months [10]. This highlights the urgent need for sustainable and context-specific IPC strategies that address the unique challenges of different healthcare settings.

Regional context

The healthcare system in this tertiary-care hospital, like other developing countries, faces challenges that contribute to an elevated risk of HAIs. Tertiary-care hospitals, such as Government Medical College (GMC), Srinagar, serve a population of over 12 million and operate under the strain of high patient volumes, with average bed occupancy rates reaching 110%, amidst persistent resource constraints. [11]. These systemic barriers, combined with a high prevalence of communicable diseases such as tuberculosis (TB; prevalence: 49 per 100,000), create conditions that favor the spread of HAIs [6]. This complex interplay of factors underscores the urgent need for regionally tailored interventions to address the specific challenges facing Kashmir's healthcare system [1,6].

Theoretical framework: bridging knowledge, attitudes, and practices

This study adopts the Knowledge-Attitude-Practice (KAP) model to analyze how HCWs’ understanding of IPC guidelines fails to translate into daily practices. The KAP framework posits that knowledge shapes attitudes, which in turn influence behavior, but this pathway is often disrupted by external factors such as resource availability, workload pressures, and institutional culture. For example, even HCWs who recognize the importance of hand hygiene may neglect it during emergencies due to time constraints. In resource-limited settings like this hospital, systemic barriers (e.g., intermittent PPE supplies) and hierarchical workplace dynamics (e.g., junior staff mirroring non-compliant seniors) further distort this pathway. By applying this model, the study identifies not only gaps in knowledge but also systemic and behavioral barriers to compliance, offering a holistic view of IPC challenges.

Research gaps and need for region-specific studies

While research on IPC in LMICs has increased, significant gaps in knowledge persist. A disproportionate number of studies concentrate on ICUs or specific infections, such as ventilator-associated pneumonia (VAP), leaving a gap in understanding hospital-wide IPC practices [3,12]. In this hospital, there is a lack of research evaluating IPC compliance across the diverse roles within healthcare settings, including doctors, nurses, and support staff (such as attendants, sanitation/cleaning workers, and waste handlers involved in hospital operations and hygiene), as well as across different departments, such as emergency rooms and general wards [13]. Furthermore, existing data predates the COVID-19 pandemic, failing to capture the significant impact of the pandemic on IPC priorities and practices. For example, a 2023 multi-center study in South India revealed improved mask compliance but stagnant hand hygiene rates post-pandemic. This highlights the need for longitudinal, region-specific analyses to understand the long-term effects of the pandemic and to develop targeted interventions that address the unique challenges of each setting [6,14].

Study objectives and scope

This cross-sectional study at GMC, Srinagar, had four primary objectives. First, it aimed to evaluate HCWs' understanding of essential IPC protocols, such as hand hygiene, PPE use, and waste management, and assess how well this knowledge aligns with global guidelines [1,7]. Second, it sought to measure HCW compliance with key IPC practices, including handwashing, sanitizer use, and mask-wearing, across different hospital departments such as the ICU, general wards, and emergency room [3,6]. Third, it set out to identify barriers that hinder consistent IPC adherence, focusing on factors like resource limitations, workload pressures, training gaps, and cultural norms [4,8]. Finally, the study compared self-reported IPC adherence before and during the COVID-19 pandemic to evaluate whether the pandemic produced sustained improvements in IPC behavior [9]. By addressing these objectives in a cohort of 300 HCWs, including doctors, paramedics, and support staff, the study aimed to provide a focused understanding of IPC knowledge, practice, and challenges in a resource-limited tertiary-care setting.

Significance and contribution

This study makes a significant contribution by providing the department-level analysis of IPC practices within the tertiary care hospitals, addressing a crucial gap in regional evidence [10,12]. The study's findings will directly inform the development of targeted interventions, such as role-specific training programs for support staff and the implementation of decentralized PPE distribution systems [7,9]. Furthermore, by connecting observed compliance patterns to the KAP framework, the study enhances our theoretical understanding of how knowledge translates into real-world practice, particularly within contexts facing systemic constraints [8,11]. On a global scale, the insights gained from this research are representative of the challenges faced by many LMICs and will assist policymakers in designing adaptable and effective IPC strategies for similar settings [13]. Ultimately, these contributions will aid in reducing HAIs, improving the safety and well-being of patients and HCWs), and strengthening the overall resilience of health systems. 

From a policy perspective, the study’s findings have the potential to shape regional and national healthcare regulations by providing empirical evidence to support mandatory IPC training modules and standardized PPE protocols. By highlighting department-specific gaps in compliance, the study advocates for tailored policy interventions that allocate resources more efficiently, ensuring that high-risk departments receive priority in PPE distribution and training initiatives. Additionally, the study underscores the importance of incorporating behavioral insights from the KAP framework into policy formulation, promoting strategies that enhance knowledge dissemination and motivation for adherence to IPC guidelines. Policymakers can leverage these findings to advocate for policy reforms that institutionalize continuous monitoring and evaluation of IPC practices, fostering a culture of safety and accountability within healthcare settings. Ultimately, the implementation of evidence-based policies inspired by this study will not only mitigate HAIs but also enhance the resilience and efficiency of health systems in LMICs, contributing to the global agenda of healthcare quality and safety.

## Materials and methods

Study setting

This cross-sectional study was conducted at the GMC in Srinagar, India, in 2022 and its associated hospitals, a major tertiary-care center serving a large population in the Kashmir region of India. These facilities include multiple clinical departments, such as general wards, ICUs (including specialized units like surgical ICU (SICU), medical ICU (MICU), and neonatal ICU (NICU)), emergency units, burn unit, operation theaters, laboratory services, outpatient clinics, and administrative/office areas, thereby offering a diverse setting for sampling HCWs on their awareness of IPC measures. This diverse setting was chosen to capture a broad representation of HCW roles and potential variations in IPC practices across different clinical contexts. HCWs from all these departments were eligible to participate in the study.

Participants and sampling

A total of 300 HCWs participated online, representing doctors (residents and consultants), paramedical staff (primarily nurses and laboratory technicians), and support staff (including ward attendants). This range of roles facilitated a comprehensive view of IPC practices at different levels of patient care. The target sample size of 300 HCWs was deemed feasible within the study's time frame and resource constraints. While a larger sample size would have been ideal, 300 participants was considered sufficient to capture a representative sample of HCWs across the diverse departments and roles within the hospital, while still allowing for detailed analysis and meaningful conclusions. All participants had been working at GMC or its associated hospitals for at least one month and were either directly or indirectly involved in patient care. Those on extended leave (e.g., maternity or sabbatical) or not engaged in patient-facing roles were not included. Inclusion criteria for participants were: (1) being an HCW (doctors, paramedics, or support staff) directly or indirectly involved in patient care at GMC Srinagar or its associated hospitals; and (2) providing informed consent to participate in the study. A non-probability convenience sampling approach was employed, with invitations extended through institutional email and common messaging platforms. Once 300 responses were received, the survey closed.

Data collection instrument

Data collection involved a structured, close-ended questionnaire that was adapted from existing IPC guidelines and relevant literature on HAIs [1]. The survey was initially piloted among 10 HCWs to assess its clarity and scope; feedback from this pilot phase was incorporated into the final version of the questionnaire to refine wording and ensure alignment with local terminology. Questions covered demographic and professional details (including age, gender, years of experience), awareness of IPC measures, vaccination status (especially hepatitis B), experiences of occupational exposure (e.g., needlestick or splash injuries), and reported adherence to IPC practices such as handwashing, mask use, and hand-sanitizer application.

Questionnaire design: rationale and methodological considerations

This cross-sectional questionnaire was designed to assess healthcare providers’ knowledge, attitudes, and practices regarding IPC within the specific context of GMC, Srinagar, and associated hospitals. A predominantly close-ended format, utilizing multiple-choice questions, was employed for several reasons: (1) it ensures standardized responses, which is essential for enhancing the reliability and comparability of the collected data; (2) it simplifies the data entry and analysis process, especially with a sample size of 300; and (3) it encourages higher response rates, as close-ended questions are generally quicker and easier to answer. Key demographic items (age, gender, profession, and specific work area within the hospital) were included to permit stratification of the data and enable subgroup analysis. This allows for the identification of potential differences in IPC awareness and practices among various demographic groups, which can inform targeted interventions. For instance, knowing if compliance differs between doctors and nurses, or between those working in the ICU versus general wards, is crucial for tailoring training and resource allocation.

Drawing on the KAP framework, the questionnaire explores these three key domains. The "Knowledge" section assesses understanding of essential IPC principles. For example, questions about the risk of transmission from various infectious agents (e.g., hepatitis B, HIV) and the effectiveness of alcohol-based hand rubs for visibly soiled hands directly gauge knowledge of core IPC concepts. The inclusion of questions about the types of precautions needed for specific infectious diseases (e.g., TB, pertussis, methicillin-resistant *Staphylococcus aureus* (MRSA)) assesses the practical application of this knowledge. Questions regarding the appropriate use of PPE), such as masks, gloves, and gowns, further explore the breadth of IPC knowledge. The "Practice" section, also relying on self-reported measures in this questionnaire, focuses on actual behaviors. Questions about hand hygiene practices (e.g., using sanitizer before/after patient contact), while self-reported, provide insights into routine behaviors. Similarly, questions about awareness of needle and sharps disposal procedures reflect an understanding of safe handling practices. While direct observation is ideal for measuring practice, self-reporting is often more feasible in large-scale surveys. Items on occupational exposures (e.g., needlestick injuries) and vaccination status (hepatitis B) are included as direct indicators of both risk and proactive health-seeking behavior. These questions provide objective measures related to IPC compliance and highlight potential vulnerabilities among HCWs.

Finally, the inclusion of questions about the impact of the COVID-19 pandemic on IPC measures allows for an understanding of how this global event may have influenced practices at both personal and institutional levels. Understanding these changes is vital for assessing the long-term impact of the pandemic on IPC behaviors. This design, by focusing on key knowledge areas, exploring potential attitudes (if added), assessing self-reported practices, and considering the impact of a major pandemic, aims to balance thoroughness with practicality, yielding robust data suitable for informing targeted interventions and policy recommendations to improve IPC compliance within the specific context of the GMC, Srinagar hospital system. A copy of the complete questionnaire is provided in the Appendices.

Data analysis

Upon completion, the data were exported from the online platform into Microsoft Excel (version 2016) for preliminary checks, including the removal of any incomplete responses. Descriptive statistics were then generated in SPSS (version 21, trial version). Categorical variables were expressed as frequencies and percentages, while continuous variables were summarized using mean and standard deviation where appropriate.

## Results

Demographic characteristics

A total of 300 HCWs participated in the study, of whom 68% (n = 204) were female and 32% (n = 96) were male. By professional designation, 62% (n = 186) were doctors, 32% (n = 96) were paramedics, and the remaining participants consisted of hospital support staff. (See Table 1 for more details about demographic breakdown). Nearly one-third of respondents worked primarily in ICUs, casualty, or triage areas, while 41% were assigned to general wards; the remaining worked in various departments throughout the hospital. Overall, 94% (n = 282) reported being actively involved in patient care on a daily basis. Hypertension was the most frequently reported comorbidity at 10% (n = 30), followed by diabetes mellitus at 4.6% (n = 14). 

**Table 1:** Knowledge and practice of IPC measures IPC: Infection prevention and control; MRSA: Methicillin-resistant *Staphylococcus aureus; *TB: Tuberculosis

Variable	Total	Percentage
Alcohol-Based Handrubs Are Not Effective if Hands Are Visibly Soiled		
Yes	237	79
No	63	21
One Should Use Hand Sanitizers Before and After Examining Patients		
Yes	297	99
No	3	1
In Biomedical Waste Segregation, Infectious Waste Is Disposed in Which Colored Bag?		
Red	75	25
Yellow	201	67
Blue	24	8
Disinfectant Used for Spill Management of Infectious Material (Blood & Body Fluids)		
Sodium hypochlorite	243	81
Glutaraldehyde	15	5
Which of the Following Types of Precaution Is Not a Transmission-Based Precaution		
Airborne	27	9
Standard	225	75
Droplet	6	2
Contact	42	14
Transmission-Based Precautions Needed for a Patient with TB		
Airborne	132	44
Contact	9	3
Droplet	144	48
Only standard	15	5
Child Admitted with Diagnosis of Pertussis. Transmission-Based Precautions Recommended		
Contact	15	5
Droplet	180	60
Airborne	48	16
Standard	57	19
Transmission-Based Precautions Required for a Patient with MRSA Wound		
Standard	99	33
Airborne	15	5
Contact	180	60
Droplet	6	2
Has COVID-19 Pandemic Improved IPC Measures?		
Yes	276	92
No	24	8

Awareness of IPC measures

When asked about overall awareness of IPC measures, 93% (n = 279) of HCWs indicated they were familiar with standard institutional guidelines. Specific awareness metrics included hand rubs (79%, n = 237), use of sanitizers (99%, n = 297), disinfectants (81%, n = 243), and standard precautions (75%, n = 225), which include the use of PPE and the appropriate disposal of sharps. Additionally, 71% (n = 213) reported being vaccinated against hepatitis B. Among those who had not received this vaccine, half cited lack of time or scheduling difficulties as the primary reason. (See Table 2 for more details.)

**Table 2:** Demographic characteristics of healthcare professionals (n = 300) SICU: Surgical ICU; MICU: Medical ICU; NICU: Neonatal ICU; IPC: Infection prevention and control

Variable	Total (Responses)	Percentage (%)
Participants (n)		
Female	198	66
Male	102	34
Age (years)		
18-44	183	61
45-59	93	31
60 & above	24	8
Profession		
Doctor	186	62
Paramedic	96	32
Administrator	9	3
Supportive staff	9	3
Work Area in Hospital		
General wards	123	41
ICU (SICU, MICU, NICU)	48	16
Casualty/Triage	45	15
Operation theater	9	3
Laboratory	45	15
Administration	30	10
Involved in Patient Care		
Yes	168	56
No	132	44
Aware About IPC Measures		
Yes	279	93
No	21	7
Source of Knowledge About IPC		
Books & articles	144	48
Internet	84	28
Seminars & lectures	72	24
Occupational Exposure to Infectious Agents		
Yes	48	16
No	252	84
Type of Exposure		
Needlestick injury	36	74
Splash injury	12	26

Compliance and practical application

Despite high levels of self-reported awareness, consistent application of IPC measures was notably lower. Although 93% of participants claimed to be aware of proper handwashing protocols, only 56% (n = 168) reported practicing handwashing consistently (i.e., before and after every patient contact). Furthermore, only 17% (n = 51) reported wearing masks consistently in applicable settings, despite the emphasis on respiratory precautions during the COVID-19 pandemic. Similarly, only 20% (n = 60) indicated they routinely supplemented or substituted handwashing with alcohol-based hand sanitizers. (See Table 2 for more details.)

Occupational exposures

A total of 16% (n = 48) of HCWs reported previous exposure to parenteral infectious agents. Of these, 74% (n = 35) were due to needlestick injuries and 26% (n = 13) were related to splash injuries (See Table 3 for more details about the breakdown). These data underscore the importance of consistent use of standard precautions, including the safe handling of sharps and the use of protective eyewear or face shields where indicated.

**Table 3:** Exposure and health characteristics of healthcare professionals COPD: Chronic obstructive pulmonary disease; IPC: Infection prevention and control; PPE: Personal protective equipment

Variable	Total (Responses)	Percentage (%)
Agent with Highest Risk of Transmission After Exposure		
Hepatitis B	174	58
HIV	93	31
Hepatitis C	33	11
Agent with Lowest Risk of Transmission After Exposure		
Hepatitis B	54	18
HIV	162	54
Hepatitis C	84	28
Vaccinated Against Hepatitis B		
Yes	213	71
No	87	29
Tested Positive for COVID-19 While Working in Hospital		
Yes, once	132	44
Yes, twice	108	36
No	60	20
Any Co-morbidity		
Hypertension	30	10
COPD	3	1
Diabetes mellitus	14	4.6
Others (heart, kidney, etc.)	4	1.4
No co-morbidity	249	83
IPC Practice Followed Rigorously in Hospital		
Hand washing	48	16
Hand sanitizers	6	2
Mask/Respirator	51	17
Full PPE	3	1
Biomedical waste disposal	0	0
Needles & sharps disposal	0	0
All of the above	192	64

Variations by departmental setting

Analysis of departmental subgroups demonstrated that while ICU, casualty, and triage staff had a higher overall awareness (96%), their reported compliance with hand hygiene measures was lower (51%) than the overall sample. In contrast, staff in general wards exhibited a marginally higher rate of consistent handwashing (58%) but a lower rate of mask use (14%) than those in critical care or emergency settings. These variations may reflect differing patient loads, perceived risk levels, or availability of resources between departments.

Summary of key observations

Overall, the majority of HCWs demonstrated a solid baseline awareness of IPC measures, especially concerning hand hygiene and the use of disinfectants. Nonetheless, there was a clear discrepancy between this awareness and the day-to-day implementation of these measures, as evidenced by suboptimal rates of handwashing and mask use. The notable incidence of parenteral exposure (especially needlestick injuries) highlights the persistent risk to HCWs and emphasizes that universal precautions are not always consistently followed. Collectively, these results point to the need for more robust educational initiatives, improved resource allocation (e.g., consistent availability of sanitizers and protective equipment), and stricter institutional policies to bridge the gap between awareness and actual practice.

## Discussion

Summary of key findings

**Figure 1:**
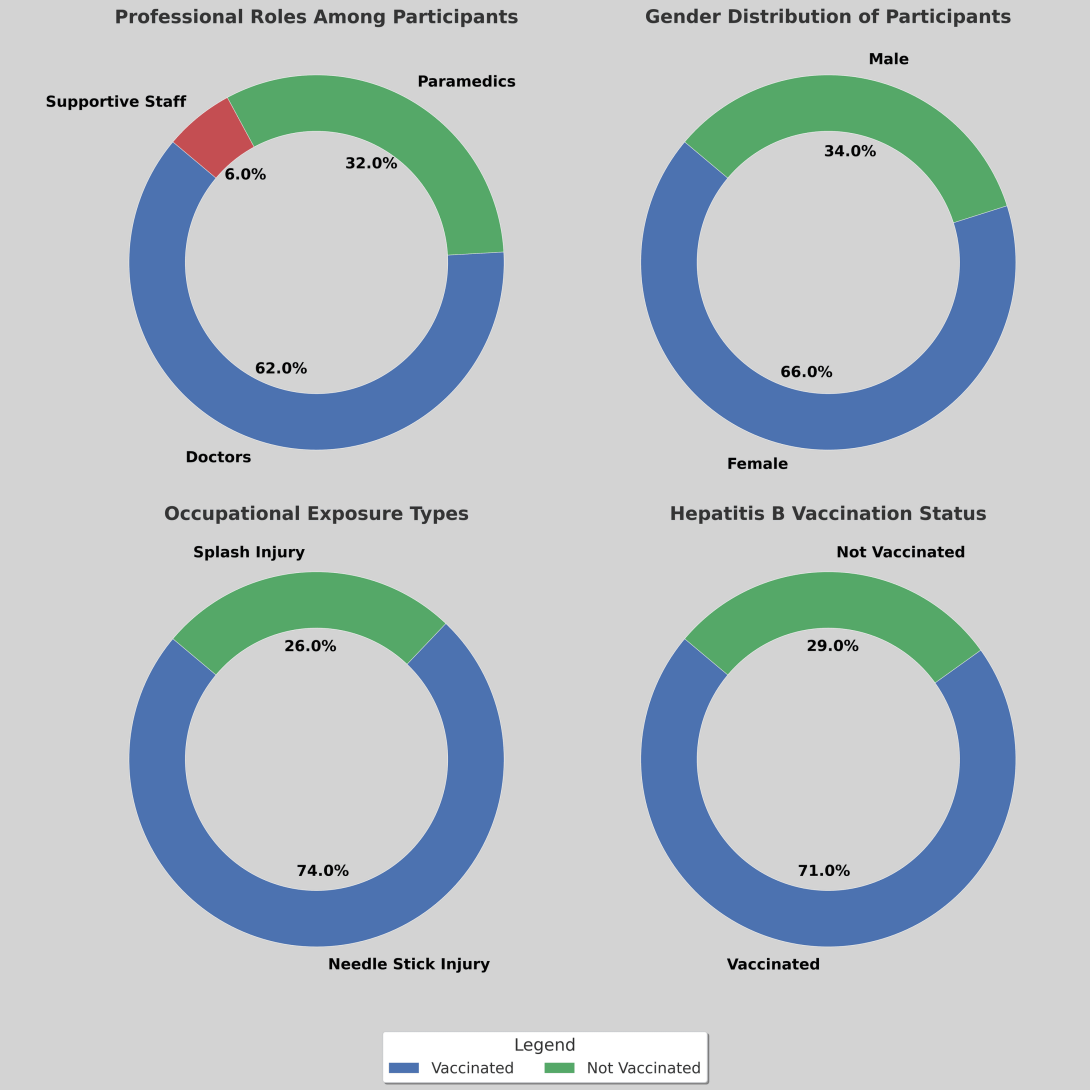
Distribution of HCWs’ professional roles (top-left), gender (top-right), occupational exposure types (bottom-left), and hepatitis B vaccination status (bottom-right) The majority of participants were doctors (62%), followed by paramedics (32%) and supportive staff (6%). Females comprised 66% of the cohort, while 34% were male. Needlestick injuries were the most common occupational exposure, and 71% of participants reported being vaccinated against hepatitis B. HCW: Healthcare worker

This study identified a significant gap between awareness and practical implementation of IPC measures among HCWs at a tertiary-care hospital. (See Figure 1 more detailed breakdown.) Despite high levels of awareness (93%), consistent adherence to IPC practices was notably low, with only 56% practicing handwashing before and after patient contact, 17% consistently wearing masks, and 20% routinely using hand sanitizers. Occupational exposures were reported by 16% of participants, predominantly due to needlestick injuries (74%), underscoring persistent safety risks. Departmental variations revealed that ICU staff had lower hand hygiene compliance (51%) compared to general ward staff (58%), reflecting the high-pressure environment of critical care settings where preventive measures are often deprioritized. Lower compliance where it should have been highest compliance is to be taken note of by hospital policy-makers and ICU in-charge. Post-pandemic mask compliance reverted to 17%, highlighting challenges in sustaining behavior change without institutional reinforcement. These findings underscore the necessity for targeted interventions that address contextual factors influencing IPC practices, particularly in resource-limited settings [15]. (See Table 1 for more details regarding this breakdown.)

Barriers to IPC compliance: a systems perspective 

This study identified four interconnected barriers to IPC compliance, contextualized within the unique challenges of this tertiary care healthcare system. 

Resource Constraints

Inconsistent Availability: Although awareness of sanitizer use was high (99%), functional dispensers may not have been available in all the wards. These findings highlight the necessity for robust supply chain management and strategic resource allocation. 

Logistical Challenges: Sanitizer dispensers were often located more than 5 m from patient beds, causing logistical delays and discouraging frequent use, and this underscores the importance of strategic placement of IPC resources [11]. 

Infrastructural Limitations: Limited access to clean water in some wards hindered handwashing, highlighting infrastructural challenges specific to the region [16]. 

Workload and Staffing Deficits: High Patient Load and Staffing Shortages

ICUs operated at 110% bed occupancy, with nurses frequently managing 8-10 patients per shift, far exceeding World Health Organization (WHO) recommendations. This overwhelming workload contributed to task-shifting, where preventive IPC practices were deprioritized in favor of urgent clinical tasks. 

Fatigue and Burnout: Extended shifts exceeding 12 hours were correlated with a 2.5-fold increase in self-reported IPC lapses, emphasizing the impact of fatigue and burnout on compliance. This aligns with global findings linking high workload to decreased compliance [14,17]. 

Behavioral Dynamics and Cultural Norms: Hierarchical Workplace Dynamics

A significant proportion (45%) of junior staff reported modeling their behaviors on senior clinicians who inconsistently adhered to IPC practices. This reflects social learning theory, where perceived social norms heavily influence behavior [18]. 

Cultural Norms and Fatalism: Cultural norms, including a degree of fatalism and normalization of non-compliance, influenced IPC behaviors. Some HCWs perceived infections as inevitable occupational risks, leading to complacency in preventive practices [7]. Addressing these dynamics requires culturally sensitive interventions promoting a culture of safety and shared responsibility [19]. 

Occupational Health Neglect: Inadequate Vaccination Coverage

29% of HCWs were unvaccinated against hepatitis B, despite the high prevalence of hepatitis B virus (2.4%) in the region. This indicates systemic neglect of occupational health and safety. 

Lack of Post-Exposure Protocols: The absence of robust post-exposure prophylaxis protocols contributed to inadequate risk management. This underscores the need for comprehensive occupational health policies that prioritize HCW safety [3,20].

Comparative analysis: bridging local and global insights

This study’s findings are consistent with global trends in IPC compliance but reveal distinct regional challenges. This tertiary-care hospital's 17% mask compliance rate reveals significant deficiencies in policy enforcement. In contrast, Kerala, India, maintained 35% compliance through consistent institutional accountability mechanisms. The comparison suggests that this hospital requires stronger policy reinforcement frameworks and more systematic monitoring to improve adherence rates. Similar declines in post-pandemic compliance were observed in Nigerian and Bangladeshi ICUs, highlighting the role of workload pressures and resource availability across low-resource settings [21]. A unique “sanitizer paradox” was identified, where near-universal awareness (99%) sharply contrasted with low usage (20%), largely due to logistical barriers, ambiguity about correct usage, and inadequate placement of sanitizer dispensers. This pattern contrasts with Bangladesh, where targeted training improved sanitizer usage to 35%, demonstrating the effectiveness of context-specific educational interventions [4,22]. This study also highlights significant hierarchical and cultural influences on IPC practices [23]. Unlike studies from high-income countries where robust institutional protocols and cultural norms enhance compliance, the findings from the tertiary-care hospital reflect complex social dynamics, including hierarchical workplace norms and fatalism, which significantly impact adherence. This underscores the need for culturally sensitive and context-specific IPC interventions.

Policy recommendations

To bridge the knowledge-practice gap and improve IPC compliance, the following multi-tiered policy recommendations are proposed:

Institutional Reforms: Digital Monitoring and Real-Time Audits 

Implement digital hand-hygiene counters and electronic monitoring systems to provide real-time feedback and enhance accountability. Real-time data analytics can identify non-compliance trends and inform targeted interventions.

Decentralized Resource Distribution: Establish decentralized PPE stations within 3 m of patient beds to minimize logistical delays and ensure consistent availability. Strategic placement of sanitizer dispensers in high-traffic areas is recommended. 

Resource Allocation and Supply Chain Management: Strengthen supply chain management systems to prevent stockouts and ensure uninterrupted availability of IPC resources, including PPE and disinfectants. Collaborate with regional suppliers to enhance supply chain resilience.

Educational Interventions - Role-Specific Training Programs: Develop department-specific training programs tailored to the unique needs of different units, especially targeting ICU staff where compliance was lowest. Simulation-based training should be integrated to demonstrate IPC feasibility under high-pressure conditions. 

Behavioral Change Communication: Utilize behavior change communication strategies, including visual cues, reminders, and posters featuring local HCWs adhering to IPC protocols. These strategies should leverage social influence to enhance adherence [16,23]. 

Continuous Education and Refresher Courses: Implement mandatory refresher courses on IPC guidelines to reinforce knowledge and address misconceptions, particularly related to hand hygiene and PPE use.

Cultural and Behavioral Interventions: Leadership Engagement and Role Modeling

Appoint “IPC champions” within departments to model compliant behaviors, influencing junior staff through positive role modeling. Senior clinicians should be actively engaged to promote a top-down approach to cultural change. 

Behavioral Nudges and Incentives: Implement behavioral nudges, such as floor markers guiding HCWs to sanitizer dispensers. Introduce incentive programs, such as recognition awards for 100% compliance, to reinforce positive behaviors. 

Occupational Health and Safety: Mandatory Vaccination and Post-Exposure Protocols

Enforce mandatory hepatitis B vaccination policies as a prerequisite for employment. Establish robust post-exposure prophylaxis protocols, including 24/7 hotlines for reporting and managing needlestick injuries. 

Mental Health and Well-Being Support: Address fatigue and burnout by implementing mental health support programs, adequate rest breaks, and flexible shift scheduling. These measures can enhance HCWs’ psychological resilience and improve IPC compliance [18].

Future directions

Future research in IPC compliance demands a more comprehensive methodological approach. A robust mixed-methods framework, combining systematic observation with in-depth qualitative investigation, would provide crucial insights beyond self-reported data. Direct observational studies could capture actual behavioral patterns and compliance rates, while focus groups and semi-structured interviews would illuminate the complex interplay of cultural norms, hierarchical dynamics, and systemic barriers that shape IPC practices [24,25].

Advanced analytical techniques could transform our understanding of compliance patterns. Machine learning algorithms and statistical modeling, particularly logistic regression and structural equation modeling (SEM), offer powerful tools for identifying key predictors of adherence and mapping the complex pathways between knowledge and practice [26]. These sophisticated analyses could reveal how various factors-from individual attitudes to organizational culture-interact to influence IPC compliance, enabling more precise and effective interventions [27].

The field would benefit significantly from well-designed longitudinal studies examining the sustainability of IPC interventions. By tracking compliance patterns and behavioral changes over extended periods, researchers could evaluate the lasting impact of various strategies, from digital monitoring systems to educational initiatives. Such longitudinal data would be invaluable in developing interventions that maintain high compliance rates over time, rather than achieving only short-term improvements [14].

Expanding research across multiple healthcare centers and diverse geographical settings is crucial for developing more generalizable and adaptable IPC strategies [28]. A coordinated network of studies spanning urban and rural facilities could identify how different resource levels, cultural contexts, and organizational structures influence compliance. These cross-regional comparisons would facilitate the development of flexible, context-sensitive guidelines that can be effectively implemented across varied healthcare environments while maintaining core safety standards [29].

Limitations

This study has several important limitations. First, the reliance on self-reported data introduces potential social desirability bias, as participants may have consciously or unconsciously overstated their adherence to IPC measures to align with perceived professional or institutional expectations [23]. Consequently, reported behaviors-particularly regarding hand hygiene and mask use-may not accurately represent actual compliance, potentially leading to an overestimation of intervention effectiveness. Although self-reporting offers practical advantages for large-scale data collection, it lacks the objectivity of direct observation or electronic monitoring, thereby introducing uncertainty and possibly obscuring the true gap between stated IPC knowledge and real-world practice.

Second, the single-center design limits the generalizability of these findings. The study was conducted in one tertiary-care hospital, whose distinctive institutional culture, infrastructure, and operational dynamics may not reflect those of other healthcare settings [13]. Variations in resource availability, workplace hierarchies, and cultural attitudes toward IPC across hospitals and regions could substantially influence compliance behaviors in ways not captured here. As such, caution should be exercised when extrapolating these results to broader or different clinical contexts, emphasizing the value of future multi-center investigations.

Finally, while the findings highlight the need for interventions such as digital compliance monitoring, decentralized PPE access, and role-specific IPC training, the study design does not permit causal inference regarding which factors most strongly predict poor adherence. In the absence of multi-variate analyses and observational validation, some conclusions remain speculative. Future research should incorporate direct observational and qualitative approaches across multiple institutions to corroborate these results and identify the most effective, context-specific strategies for improving IPC compliance and reducing HAIs.

## Conclusions

HAIs remain a pressing challenge in resource-limited healthcare settings. Our study highlights a critical gap between knowledge and practice of IPC measures: Although 93% of staff reported awareness of IPC protocols, only 56% consistently practiced hand hygiene, 17% regularly used masks, and 20% adhered to appropriate hand sanitizer use. Identified barriers included resource constraints, workforce shortages, hierarchical influences that reinforced non-compliance, and inadequate occupational health safeguards.

## Figures and Tables

**Figure 2:**
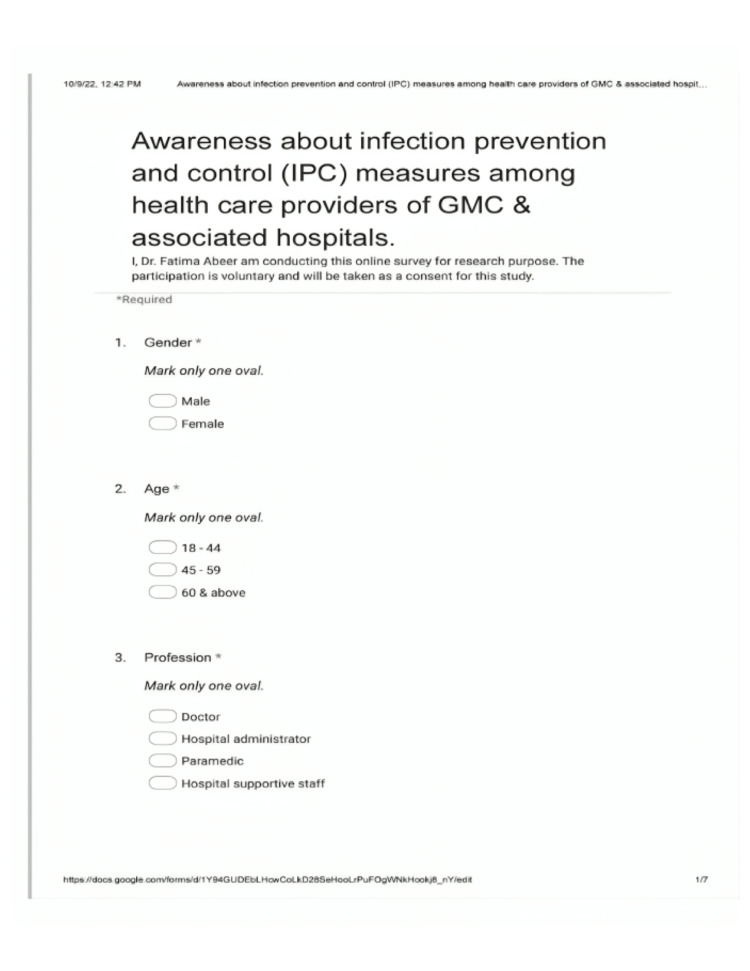
First page of the questionnaire

**Figure 3:**
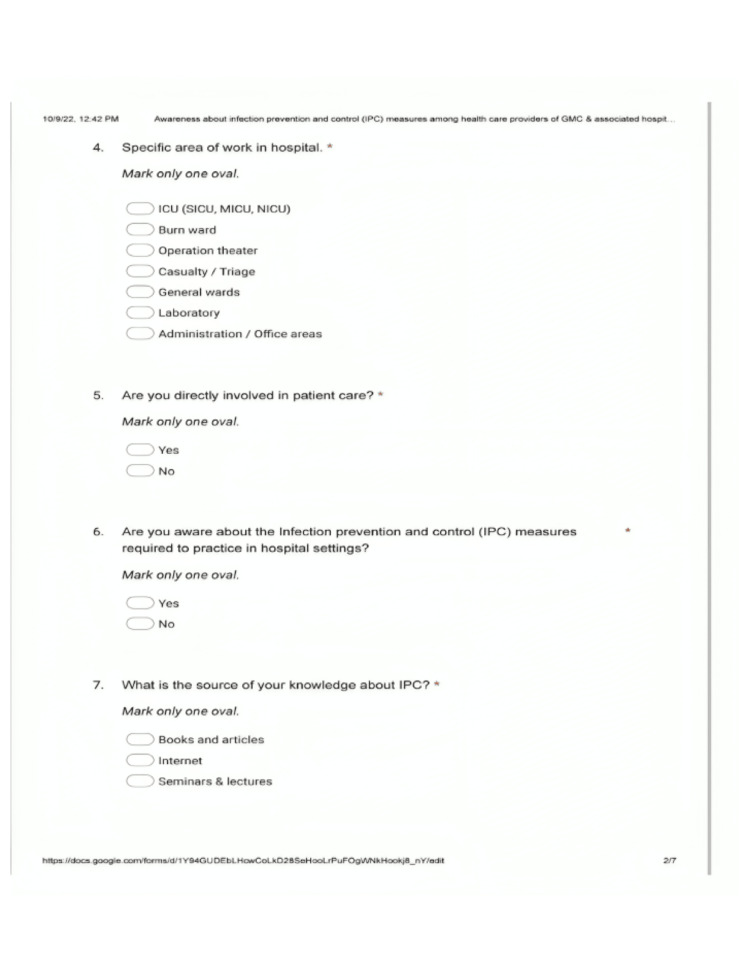
Second page of the questionnaire

**Figure 4:**
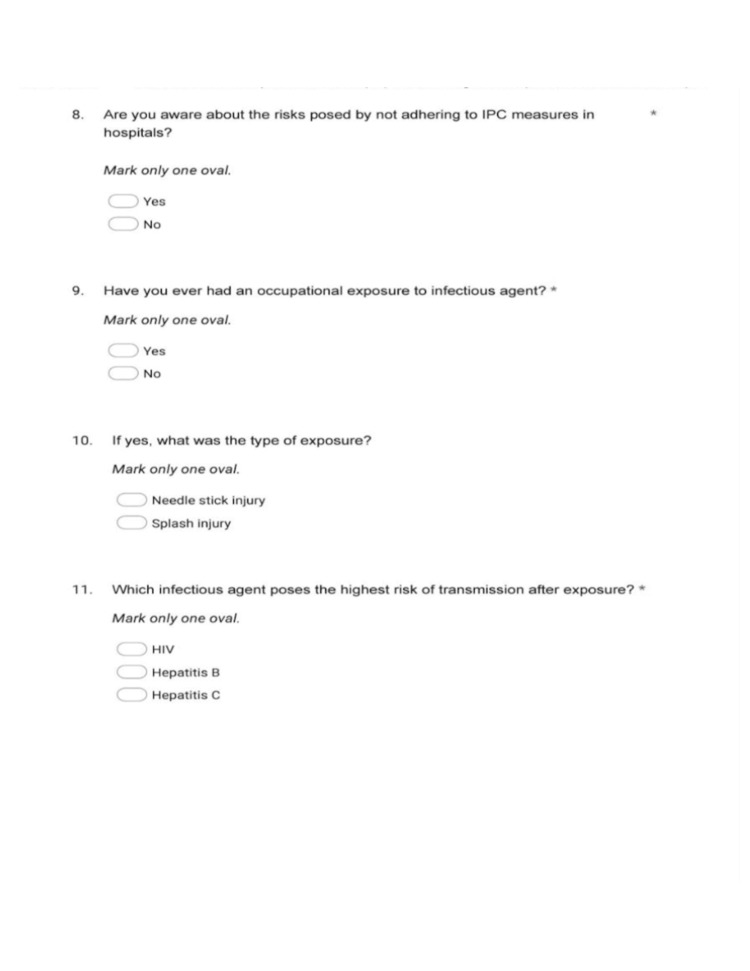
Third page of the questionnaire

**Figure 5:**
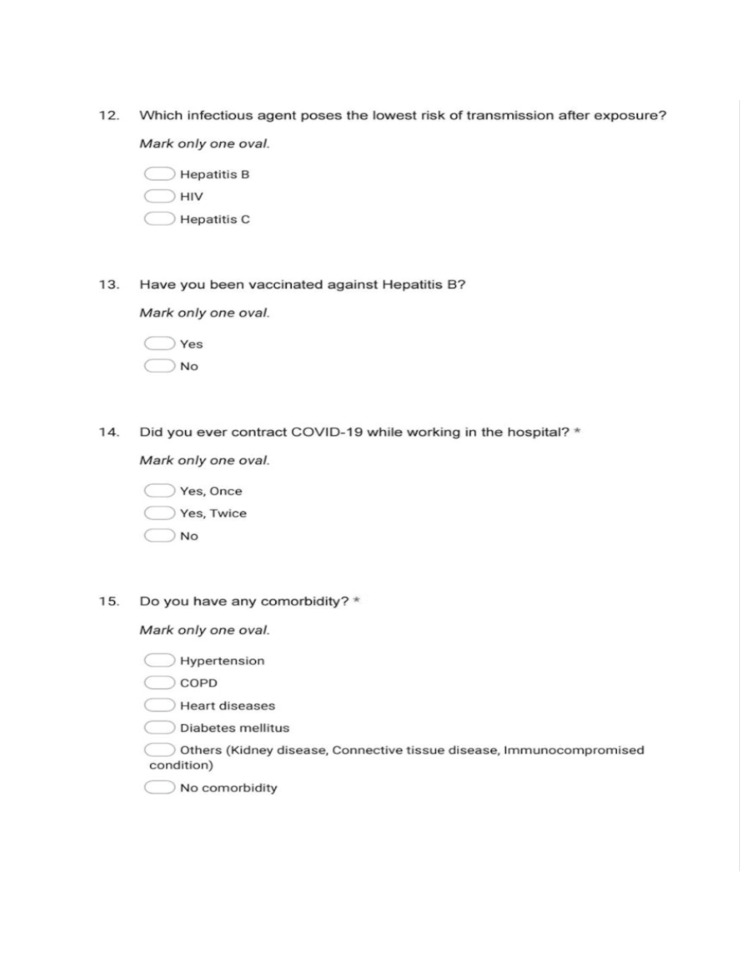
Fourth page of the questionnaire

**Figure 6:**
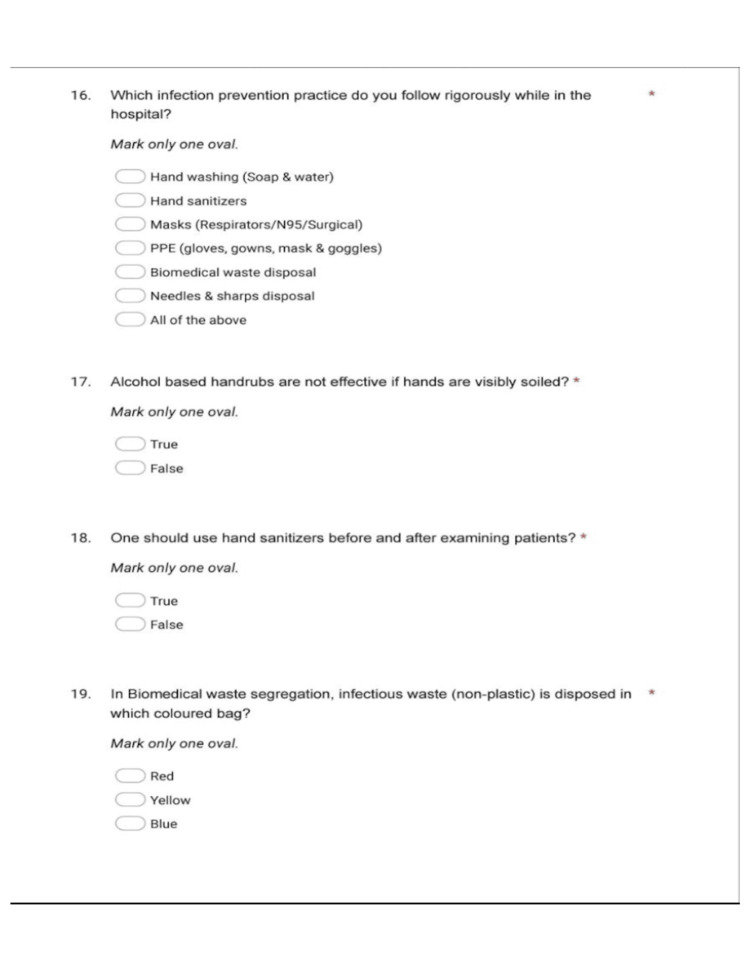
Fifth page of the questionnaire

**Figure 7:**
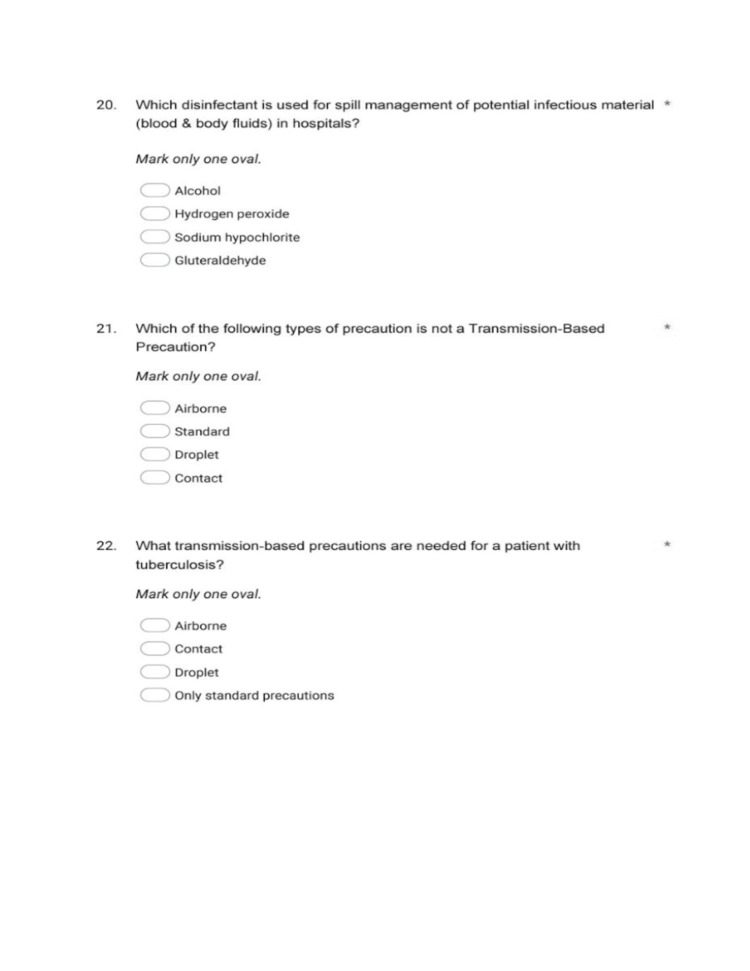
Sixth page of the questionnaire

**Figure 8:**
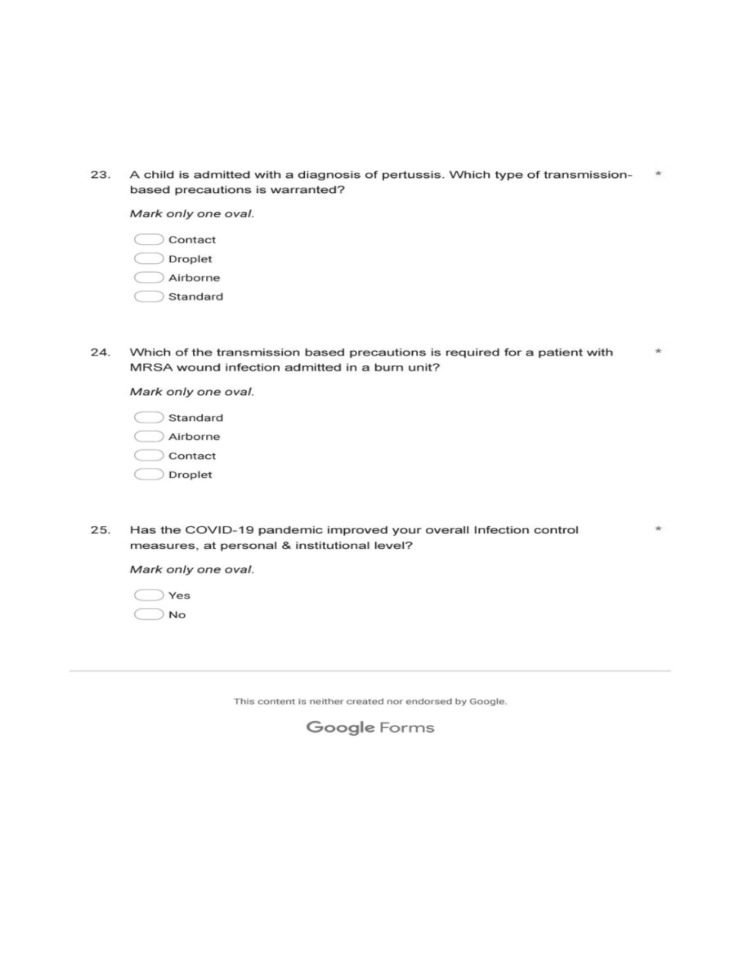
Seventh Page of the Questionnaire

## Appendices

The questionnaire used in this study was developed by our research team to measure public awareness of infection control awareness and practices among healthcare workers. Distributed via Google Forms, all questions are original content created by the authors without adapting any previously published materials. No copyright permissions were required. The instrument underwent expert review for validity and clarity before distribution. As the questionnaire is our team's intellectual property and has not been published elsewhere, we include it in this article with full permission and no conflicts of interest.
